# Ameliorating high-fat diet-induced sperm and testicular oxidative damage by micronutrient-based antioxidant intervention in rats

**DOI:** 10.1007/s00394-022-02917-9

**Published:** 2022-06-16

**Authors:** Md Mustahsan Billah, Saroj Khatiwada, Virginie Lecomte, Margaret J. Morris, Christopher A. Maloney

**Affiliations:** 1 School of Medical Sciences, UNSW Sydney, Sydney, NSW 2052 Australia; 2 School of Health Sciences, UNSW Sydney, Sydney, NSW 2052 Australia

**Keywords:** Antioxidants, Sperm, Testis, Micronutrients, Obesity, Oxidative stress

## Abstract

**Purpose:**

Emerging evidence from rodent studies suggests that high-fat-diet (HFD)-induced obesity is characterized by increased oxidative damage in sperm and testis. However, interventions using micronutrient supplementation to mitigate oxidative damage in obesity have not been extensively studied. This study aimed to investigate the effect of an antioxidant-based micronutrient supplement (added folate, vitamin B_6_, choline, betaine, and zinc) on sperm and testicular oxidative damage in HFD-fed male Sprague Dawley rats.

**Methods:**

Rats (3-weeks-old, 12/group) were weaned onto control (C) or HFD (H) or these diets with micronutrient supplement (CS; HS); sperm and testis were harvested at 30.5 weeks. To assess oxidative stress and antioxidant capacity in testis, levels of malondialdehyde (MDA), glutathione (GSH), folate and susceptibility index (SI) of pro-oxidative damage, mRNA expression of Nrf2, NFκB-p65, IL-6, IL-10 and TNF-α, in addition to superoxide-dismutase (SOD), catalase and glutathione-peroxidase (GPx) activities were measured. 8-hydroxy-2-deoxyguanosine (8-OHdG) were assessed in both sperm and testis.

**Results:**

HFD-fed rats had significantly increased 8-OHdG content in sperm and testis, increased testicular SI, decreased testicular weight, SOD and GPx activity compared to control. Strikingly, supplementation of HFD appeared to significantly reduce 8-OHdG in sperm and testis (22% and 24.3%, respectively), reduce testicular SI and MDA content (28% and 40%, respectively), increase testicular weight (24%), SOD and GPX activity (30% and 70%, respectively) and GSH content (19%). Moreover, supplementation had significant impact to increase testicular folate content regardless of diet. Furthermore, an overall effect of supplementation to increase testicular mRNA expression of Nrf2 was observed across groups. Interestingly, testicular SI was positively correlated with sperm and testicular 8-OHdG and MDA content, suggesting a critical role of testicular antioxidant activity to combat oxidative damage in sperm and testis.

**Conclusion:**

Our findings suggest that antioxidant-based micronutrient supplement has the potential to interrupt HFD-induced sperm and testicular oxidative damage by improving testicular antioxidant capacity.

**Graphical abstract:**

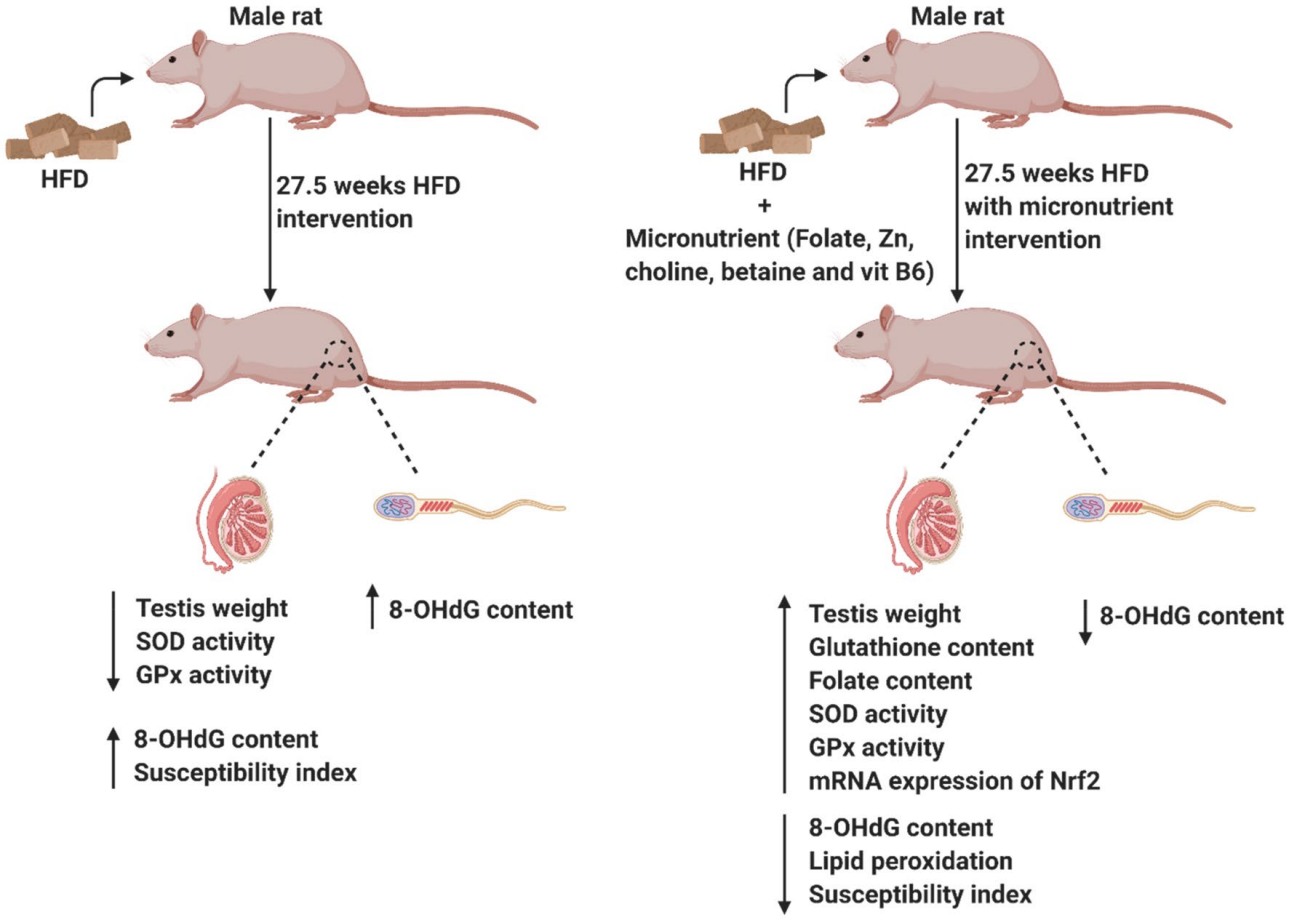

**Supplementary Information:**

The online version contains supplementary material available at 10.1007/s00394-022-02917-9.

## Introduction

The prevalence of obesity, a major public health challenge across the world, has substantially increased in adults over the last 40 years [1]. In developed countries including USA, UK and Australia, males are more affected by obesity than females [2]. Obesity in male rodents is closely linked with sperm and testicular oxidative damage, characterized by increased lipid peroxidation [3, 4], reactive oxygen species (ROS) production and oxidative DNA damage [3, 5, 6]. Sperm oxidative DNA damage is increasingly being considered as one of the potential mechanisms underpinning paternal programming of offspring health in both human and rodents [7, 8]. Thus, reduction of oxidative stress by interventions could be a potential approach to mitigate obesity-induced sperm and testicular oxidative damage and associated programming.

Micronutrients are generally consumed in small amounts in the diet; however, they are essential and play critical roles as antioxidants [9]. A growing list of studies suggest that obesity is associated with decreased systemic levels of micronutrients having antioxidant property particularly folate, zinc, choline, betaine and vitamin B_6_ [10–14]. Such deficiencies may lead to increased oxidative stress [15], development of comorbidities [16] and decreased sperm quality [17]. Moreover, obesity-induced impaired antioxidant capacity can further accelerate testicular and sperm oxidative damage as demonstrated in rodent studies [18, 19]. On the other hand, micronutrient supplements have been found to not only improve conventional sperm parameters (e.g., sperm count, motility, morphology) but also to reduce ROS production, DNA damage, increase antioxidant capacity and improve DNA integrity of sperm in sub-fertile, and infertile men [20–25] as well as infertile [26] and undernourished [27] rodents. However, evidence supporting micronutrient interventions to attenuate obesity-induced oxidative stress in the male reproductive system is limited. To date, there has been only one study in rodents investigating the potential impacts of micronutrient supplement in ameliorating obesity-induced oxidative DNA damage in sperm [5]. Moreover, no study in rodents has investigated the effects of micronutrient supplement on obesity-induced oxidative DNA damage in testis. Given the limited information in the context of obesity-induced sperm/testicular oxidative damage, such micronutrient intervention requires further attention.

Therefore, this study aimed to investigate whether micronutrient supplementation in Sprague Dawley male rats could ameliorate HFD-induced oxidative damage in testis and sperm. The metabolic assessment of these animals was reported recently [28]. Semisynthetic control or HFD were supplemented with a unique formulation comprising five micronutrients: folate, vitamin B_6_, choline, betaine, and zinc (ZnSO_4_) as previously described [28]. These components participate in one carbon metabolism either as a substrate or a cofactor [29], and can also act as antioxidants [30]. One carbon-metabolism is a set of pathways where one-carbon moieties are transferred from donors to intermediate carriers and finally used for critical cellular process including the production of the antioxidant glutathione [31]. The addition of vitamin B_6_ in this formulation was intended to increase glutathione (a major intracellular antioxidant) production via the trans sulphuration pathway [32] with a view to combat oxidative attack more efficiently. Very little is known about the benefits of supplementing multiple micronutrients that participate in one-carbon metabolism. As far we are aware, only folate and zinc (out of the five components of our micronutrient formulation) have been tested previously in rodents to target HFD-induced oxidative damage in sperm [5]. Moreover, to the best of our knowledge, the impact of increased vitamin B_6_, combined with other micronutrients used here in the context of HFD-induced sperm and testicular oxidative damage is unknown. We hypothesized that improving testicular antioxidant content/activity through micronutrient supplementation could counteract testicular and sperm oxidative stress in HFD-fed rats. Interestingly significant beneficial effects of micronutrient supplement on sperm and testicular oxidative stress were seen.

## Materials and methods

### Animals, diets and micronutrient supplement

**Fig. 1:**
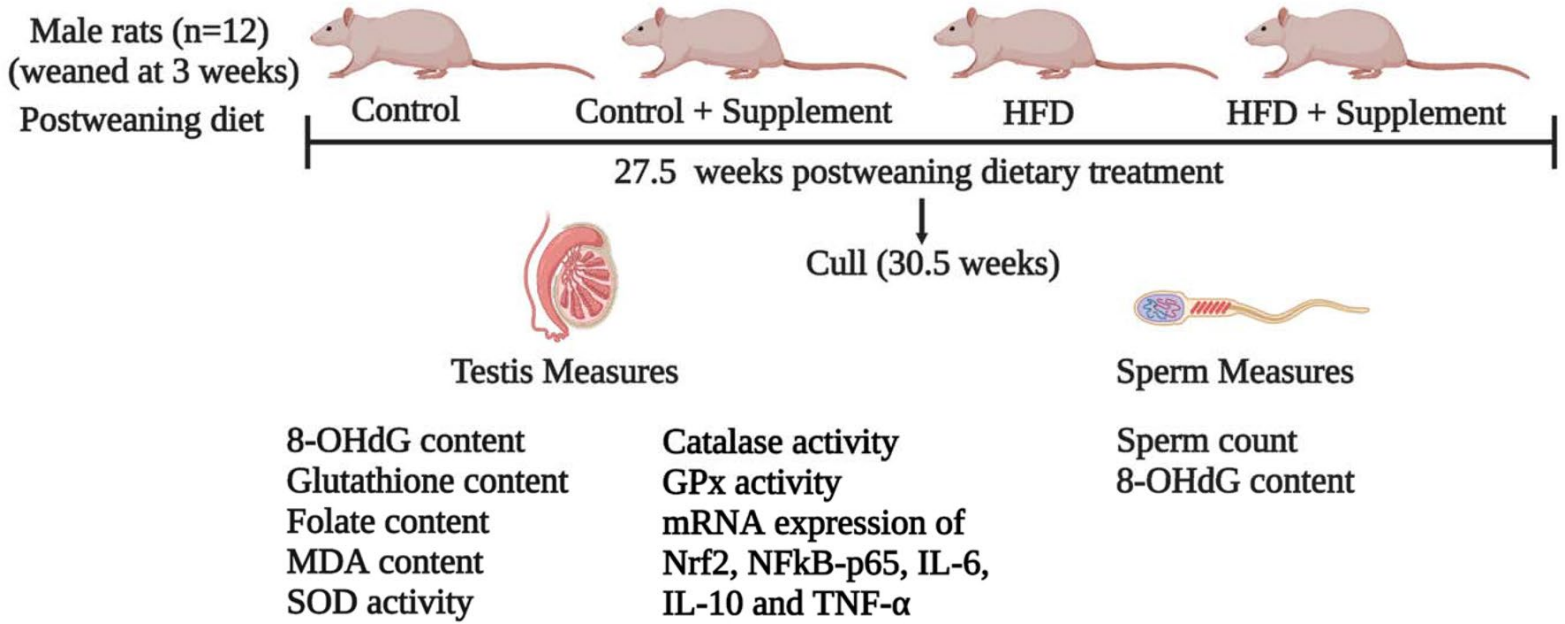
Experimental design and timeline. Male Sprague Dawley rats were fed control or HFD with or without supplement from weaning till 30.5 weeks of age. At cull (30.5 weeks), sperm was harvested and analysed for sperm count and 8-OHdG content and testis was analysed for 8-OHdG, MDA, folate and GSH content, the activity of SOD, catalase and GPx, susceptibility index of pro-oxidative damage and mRNA expression of Nrf2, NFκB-p65, IL-6, IL-10 and TNF-α. *8-OHdG* 8 hydroxy-2-deoxyguanosine, *MDA* malondialdehyde, *SOD* superoxide-dismutase, *GPx* glutathione-peroxidase, *GSH* glutathione, *Nrf2* nuclear factor erythroid 2-related factor 2, *NFκB-p65* nuclear factor kappa-light-chain-enhancer of activated B cells, subunit p65, *IL-6* interleukin-6, *IL-10* interleukin-10, *TNF-α* tumor necrosis factor alpha

Male Sprague Dawley rat pups generated in the Biological Resources Centre, UNSW SYDNEY (Founders sourced from ARC, Perth, WA) were maintained at 21 ± 2 °C; 12:12 h light/dark condition. The use of animals and all experimental procedures were approved by the Animal Ethics Committee of UNSW SYDNEY (Ethics ID: 17/27A). From 3 weeks of age, animals were housed 2–3 rats per cage and randomly allocated to four diet groups (*N* = 12) until cull. The experimental design is depicted in Fig. 1. The diets (Table 1) used were control diet (denoted by C), control diet containing micronutrient supplement (denoted by CS), HFD (denoted by H) and HFD-containing micronutrient supplement (denoted by HS). To maintain a body weight difference between control and HFD-fed animals, we limited the energy intake of the C and CS groups from 7 weeks of age (from 4 weeks of diet) to 407 kJ (32 g/rat/day of diet), which corresponds to the daily recommended intake for rats. Our extensive experience with adult SD male rats suggests this energy intake is sufficient for normal growth [33]. The metabolic and liver outcomes from these animals was published recently [28].

**Table 1:** Chemical composition per kilogram of Control (C,) HFD (H), Control supplemented (CS) and HFD supplemented (HS) diets

Ingredients (g/kg)	C	H	CS	HS
Casein	140	140	140	140
Sucrose	100	405	100	405
Canola Oil	40	50	40	50
Lard		190		190
Cellulose	50	50	50	50
Corn starch	620	115	620	115
dl Methionine	3	3	3	3
Mineral mix (AIN93M)	35	35	35	35
Vitamin mix (AIN93)	10	10	10	10
Choline bitartrate^a^	4.1	4.1	36.5	36.5
Betaine^a^			15	15
Folic acid^a^			0.013	0.013
Pyridoxine HCl (Vit B_6_)^a^			0.0295	0.0295
ZnSO_4_.H_2_O^a^			0.296	0.296

The supplemented diets had additional Choline, Betaine, Folic acid, Vitamin B_6_ and Zinc

^a^Additional to AIN93M vitamin mix

The diets were prepared in-house using commercial ingredients, and the micronutrient supplements were added during preparation. The composition of both C and HFD were similar except for sucrose, canola oil, lard, corn starch and supplemented micronutrients content as published recently [28]. Control diet and HFD were prepared based on American Institute of Nutrition 93 G/M (AIN 93 G/M) formulation [34], and SF03-020 (Specialty Feeds, Western Australia), containing 23% total fat and 20 MJ/kg digestible energy, respectively. The energy content of our control and control supplemented diet was 12,720 kJ/kg diet, and that of HFD and HFD supplemented diet was 20,439 kJ/kg diet. The micronutrient formulation for the supplementation was designed based on the Wolff 3SMZ diet used in mice except the increase in vitamin B_12_ was replaced by additional B_6_ and methionine was given at normal levels [35].

### Testis, sperm collection and sperm count

Rats were euthanised at 30.5 weeks of age to collect sperm and testis samples for further assays and all samples were stored at −80 ºC until analysed. Testis was powdered under liquid nitrogen for use in assays described below. Sperm was collected from both cauda of male rats using a swim out method. Several pores were made in the cauda (using 23 G needle) kept in Biggers, Whitter and Whittingham (BWW) buffer with underlying hydrated mineral oil in a petri dish to collect motile sperm. The plate was incubated for 10 min at 37 ºC. After that, the BWW containing sperm was aspirated and aliquoted into two tubes and snap frozen for further experiment. Sperm count was determined by placing 10 µl of 1 in 20 diluted sample in a hemocytometer, placing under a microscope (Olympus CH-2, Japan, 100X magnification); number of sperm counted in five squares were then multiplied by 10^6^ to get counts per ml.

### Oxidative DNA damage (8-OHdG) measurement in sperm and testis

At first, DNA was extracted from sperm and powdered testis following Qiagen DNeasy Blood and Tissue kit protocol (Cat No: 69504). 1–2 μg DNA was then treated with nuclease P1 and alkaline phosphatase following New England BioLabs protocols (Cat: M0660S and Cat: M0371S, respectively) to convert DNA samples into single nucleosides. 8-OHdG level in the extracted single nucleoside samples was determined using 8-hydroxy 2 deoxyguanosine ELISA kit as per the manufacturer’s instructions (Abcam Cat ID: ab201734). The 8-OHdG concentration in each sample was calculated by five-parameter logistic regression interpolated from the standard curve using elisaanalysis.com software. The intra- and inter-assay precision CVs for this assay in sperm and testis were ≤ 5% and ≤ 6%, respectively.

### Testicular folate and lipid peroxidation measurement

50–100 mg of powdered testis was homogenized in phosphate buffered saline (PBS) to measure folate and lipid peroxidation content. In the supernatant, folate concentration and lipid peroxidation (Malondialdehyde, MDA) content were determined by an ELISA (Monobind Inc., Cat: 7825-300B, CA, USA) and colorimetric assay (OxiSelect™ TBARS Assay Kit, Cat: STA-330, Cell Biolab Inc), respectively, as per the manufacturer’s protocols. Values were normalized to the amount of tissue extracted. The intra- and inter-assay precision CVs for folate and lipid peroxidation assay in testis were ≤ 7% and ≤ 6%, respectively.

### Testicular glutathione content measurement

To measure total glutathione content, 50–100 mg of powdered testis was homogenized using 5% sulphosalicylic acid (SSA). Total glutathione concentration was estimated in the supernatant by kinetic assay using Ellman’s reagent kit (Sigma-Aldrich, Cat: EIAGHSC, St. Louis, MO, USA) following the manufacturer’s protocol. The value was normalized to the amount of tissue used to prepare extract. The intra- and inter-assay precision CVs for this assay were ≤ 8%.

### Testicular superoxide-dismutase (SOD) activity measurement

Powdered testis (~ 100 mg) was homogenised in 1 mL cold 20 mM HEPES buffer (pH 7.2 containing 1 mM EGTA, 210 mM mannitol and 70 mM sucrose). SOD activity was then measured in the collected supernatant following SOD assay kit protocol as per the manufacturer’s instructions (Cayman Chemical, Cat: 706002, MI, USA). The intra- and inter-assay precision CVs for this assay were ≤ 5%.

### Testicular catalase activity measurement

Powdered testis (~ 100 mg) was homogenised in 1 mL cold 50 mM potassium phosphate (pH 7.0 containing 1 mM EDTA). Catalase activity was then measured in the collected supernatant following a Catalase assay kit protocol (Cayman Chemical, Cat: 707,002, MI, USA); intra- and inter-assay precision CVs were ≤ 8%.

### Testicular glutathione-peroxidase (GPx) activity measurement

One mL cold 50 mM Tris–HCl (pH 7.5, 5 mM EDTA and 1 mM DTT) was used to homogenize 100 mg powdered testis. The collected supernatant was then assayed for GPx activity using Glutathione peroxidase assay kit protocol (Cayman Chemical, Cat: 703102, MI, USA) as per the manufacturer’s protocol. The intra- and inter-assay precision CVs for this assay were ≤ 6%.

### Susceptibility index of pro-oxidative damage

Susceptibility index of pro-oxidative damage in testis was calculated from the ratio of SOD to catalase plus GPx activity as described elsewhere [36]. The amount of hydrogen peroxide (H_2_O_2_) produced upon dismutation of superoxide by SOD needs to be converted to a non-toxic product by catalase and GPx antioxidant. If the catalase and GPx activity is reduced, the produced H_2_O_2_ starts accumulating and leads to further oxidative damage in biological systems [37]. Therefore, a higher susceptibility index reflects increased oxidative damage.

### Testicular RNA extraction and measurement of gene expression

Total RNA was extracted from testis, and cDNA was synthesized as described previously [38]. Real-time qPCR was performed on the QuantStudio 12K Flex (Life Technologies, CA, USA) as described previously [38], and in compliance with the MIQE guidelines [39]. The stability of each reference gene was analysed using a web based comprehensive tool RefFinder (https://www.heartcure.com.au/reffinder/), and the best combination of genes (Gapdh and TBP) was selected. Expression of genes involved in oxidative stress (Nrf2) and inflammation (NFκB-p65, IL6, IL10 and TNF-α) and the reference genes was measured using TaqMan RT-PCR. Relative gene expression was calculated using the 2^−ΔΔ*C*T^ method, normalised against two reference genes and an external calibrator using pooled sample [40]. Genes selected for TaqMan RT-PCR and the corresponding hydrolysis probe references (Life Technologies, CA, USA) are listed in Supplementary Table 1.

### Statistical analysis

Statistical analyses were performed using IBM SPSS v 23.0 software. Normally distributed data (analysed by Shapiro–Wilk normality test) were expressed as mean ± SEM. Data were analysed by two-way ANOVA. The main effects followed by Tukey HSD post hoc were considered if there were no interaction effects. In case of interaction between independent factors, simple main effect analysis was performed, and the resulting post hoc from interaction effect was considered. Associations of testicular susceptibility index with other oxidative stress parameters in sperm and testis were determined by Pearson correlation where appropriate.

Overall HFD and supplement effect are denoted by ‘$’, and ‘#’, respectively, where required. Interactions between HFD and supplement are noted when significant. Differences were considered significant at *p* < 0.05.

## Results

### Effect of HFD and supplementation on body weight, fat mass, testis weight and sperm count at cull (30.5 weeks)

The overall growth and energy intake including the metabolic outcomes of these animals has been reported previously [28]. In summary, the weekly energy intake of H and HS groups were significantly higher than C and CS groups (*p* < 0.001 for both comparisons), respectively. There were no differences in energy intake among H and HS-fed animals. At weaning, body weight across the groups was similar. As expected, HFD promoted weight gain (*p* < 0.001, H vs C), and supplemented HFD-fed rats had significantly reduced body weight (*p* < 0.001, HS vs H) despite their similar energy intake throughout the study [28]. At cull, a significant interaction effect of HFD and supplement was observed on body weight, absolute and relative retroperitoneal fat mass and relative testis weight indicating that HFD-fed rats had increased body weight (H vs C, ~ 21%, *p* < 0.001), absolute fat mass (H vs C, 139%, *p* < 0.001), relative fat mass (H vs C, 96%, *p* < 0.001; HS vs CS, ~ 48%, *p* < 0.05) and decreased relative testicular weight (H vs C, ~ 17%, *p* < 0.001) compared to C group (Table 2). The supplement appeared to normalise those parameters in the HS group (HS < H, 21.4%, *p* < 0.001 for body weight; HS < H; ~ 49%, *p* < 0.001 for absolute fat mass; HS < H, 35.2%, *p* < 0.001 for relative fat mass and HS > H, ~ 24%, *p* < 0.001 for relative testis weight) as measured by simple main effect (Table 2). Feeding HFD and supplement had no significant impact on the sperm count (Table 2).

**Table 2:** Body weight, fat mass, testicular weight and sperm count at cull

Parameters	C	CS	H	HS	HFD effect	Supplement effect	Interaction effect
Body weight (g)	632.5 ± 15.1	613.0 ± 8.7	765.0 ± 16.6^a^	601.4 ± 12.9.^d^	** < 0.001**	** < 0.001**	** < 0.001**
RpWAT (g)	6.77 ± 0.28	5.64 ± 0.41	16.20 ± 1.28^a^	8.32 ± 0.93.^d^	** < 0.001**	** < 0.001**	** < 0.001**
RpWAT (% body weight)	1.07 ± 0.04	0.92 ± 0.06	2.10 ± 0.14^a^	1.36 ± 0.13^b,d^	** < 0.001**	** < 0.001**	** < 0.001**
Testis weight (% body weight)	0.60 ± 0.02	0.64 ± 0.01	0.50 ± 0.01^a^	0.62 ± 0.02.^d^	**0.001**	** < 0.001**	**0.011**
Sperm count (million/mL)	17.5 ± 1.3	18.1 ± 2.9	16.5 ± 2.5	17.6 ± 1.5	0.745	0.726	0.925

Data are shown as mean ± SEM (n = 11–12 per group). Effects of HFD and supplement were assessed by simple main effect analysis, and the resulting post hoc was considered. Post hoc HFD effect ‘a’ (H vs C), ‘b’ (HS vs CS) and supplement effect ‘d’ (HS vs H). Differences were considered significant at *p* < 0.05 (Bolded)

*C* control diet, *CS* control diet with supplement, *H* HFD, *HS* HFD with supplement

### Effect of HFD and supplementation on oxidative DNA damage in sperm and testis

**Fig. 2:**
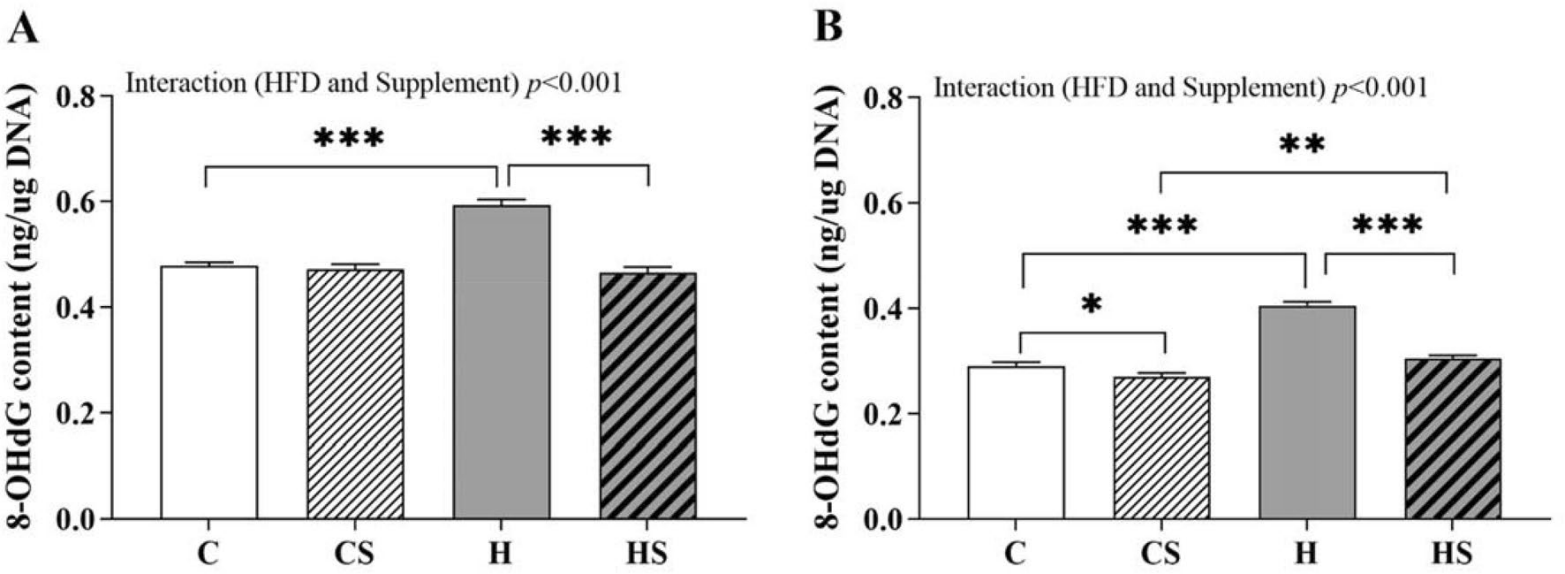
**A** Sperm and **B** testicular 8-OHdG content. Data are shown as mean ± SEM (n = 11–12 per group). Effects of HFD and supplement were assessed by simple main effect analysis, and the resulting post hoc was considered. HFD and supplement interaction effect *p* < 0.001. *C* Control diet, *CS* control diet with supplement, *H* HFD, *HS* HFD with supplement, *8-OHdG* 8 hydroxy-2-deoxyguanosine. *p < 0.05, **p < 0.01 and ***p < 0.001

Both HFD and supplementation had overall significant effect (*p* < 0.001) on sperm and testicular oxidative DNA damage (8-OHdG) along with a significant interaction effect (*p* < 0.001) (Fig. 2A, B). HFD-fed rats had 24% increased 8-OHdG content in their sperm (H vs C, *p* < 0.001; Fig. 2A) and 13–40% increased 8-OHdG content in testis (H vs C, ~ 40%, *p* < 0.001; HS vs CS, ~ 13.3%, *p* < 0.01; Fig. 2B) as analysed by simple main effect. This effect was normalized in both sperm (HS < H, ~ 22%, *p* < 0.001; Fig. 2A) and testis (HS < H, ~ 25%, *p* < 0.001; Fig. 2B) by supplementation in HS rats. Supplementation combined with control diet significantly reduced 7.2% 8-OHdG content in testis (CS vs C, *p* < 0.05; Fig. 2B) but not in sperm (CS < C; ~ 1.5%, *p* > 0.05; Fig. 2A).

### Effect of HFD and supplementation on testicular lipid peroxidation and folate content

**Fig. 3:**
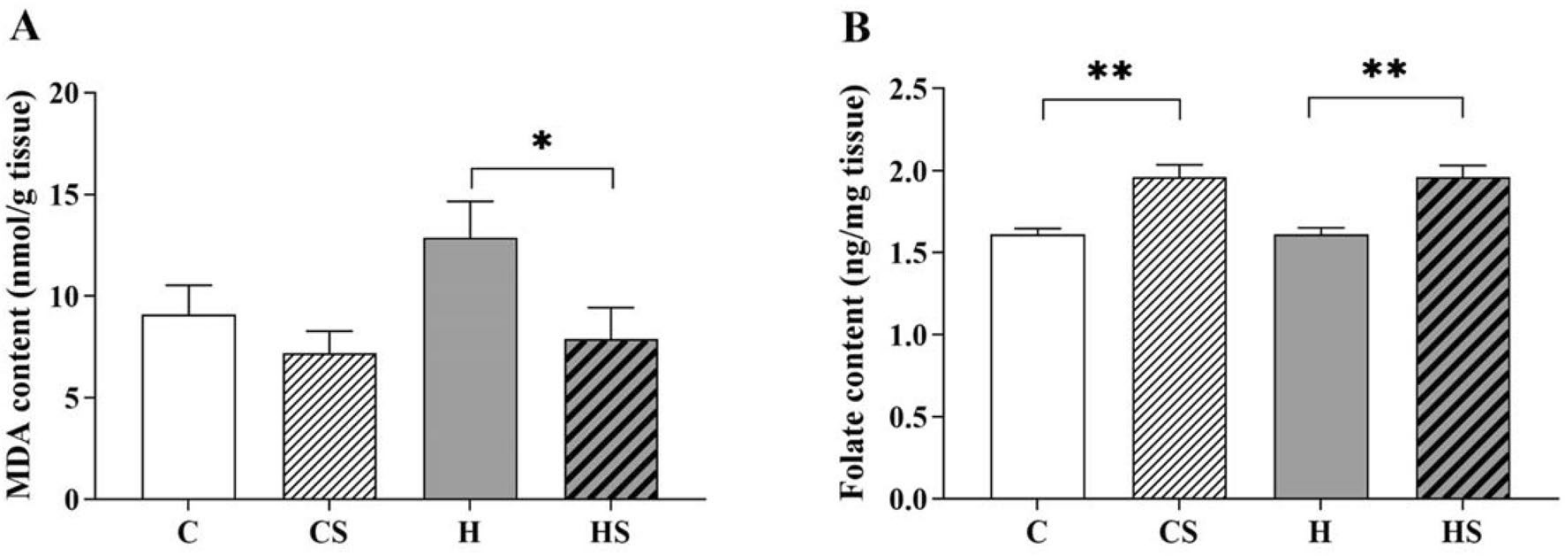
Testicular **A** MDA and **B** folate level. Data are shown as mean ± SEM (*n* = 11–12 per group). Effects of HFD and supplement were assessed by two-way ANOVA followed by Tukey HSD post hoc (Folate) or LSD post hoc (MDA). Overall HFD effect *p* > 0.05 (MDA and folate), supplement effect *p* = 0.027 (MDA) and *p* < 0.001 (folate). *C* control diet, *CS* control diet with supplement, *H* HFD, *HS* HFD with supplement, *MDA* malondialdehyde. *p < 0.05 and **p < 0.01

In testis, HFD intake did not significantly affect lipid peroxidation (manifested as MDA, Fig. 3A) and folate content (Fig. 3B). Interestingly, supplementation in HFD-fed rats had an impact to significantly decrease testicular MDA content by 40% compared to H group (*p* = 0.02, Fig. 3A). Supplement when combined with control diet did not appear to affect testicular MDA content (CS < C, ~ 21%, *p* = 0.3; Fig. 3A). An overall significant effect (*p* < 0.001) of supplement on testicular folate content demonstrated that supplemented groups regardless of their diet had increased folate content compared to non-supplemented groups (CS vs C, HS vs H, ~ 22%, *p* = 0.001; Fig. 3B).

### Effect of HFD and supplementation on testicular antioxidant capacity

**Fig. 4:**
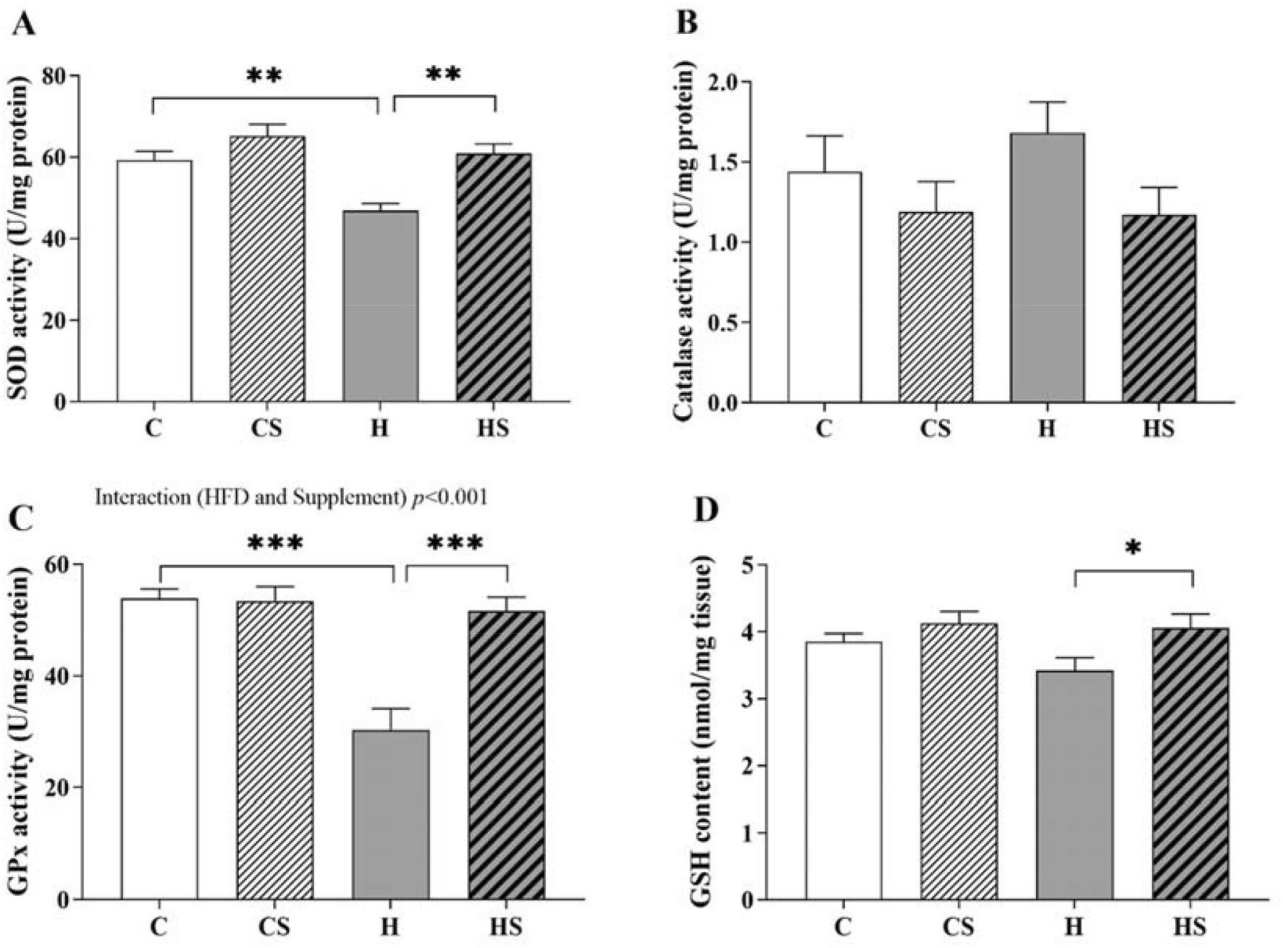
Testicular activity of **A** SOD, **B** catalase, **C** GPx and **D** GSH content. Data are shown as mean ± SEM (n = 11–12 per group). Effects of HFD and supplement were assessed by two-way ANOVA followed by Tukey HSD post hoc (SOD) or LSD post hoc (GSH) or by simple main effect analysis, and the resulting post hoc was considered (GPx). Overall HFD effect *p* = 0.001 and supplement effect *p* < 0.001 (SOD), *p* > 0.05 (catalase), overall supplement effect *p* = 0.013 (GSH), HFD and supplement interaction effect *p* < 0.001 (GPx). *C* control diet, *CS* control diet with supplement, *H* HFD, *HS* HFD with supplement, *SOD* superoxide-dismutase, *GPx* glutathione-peroxidase, *GSH* glutathione. *p < 0.05, **p < 0.01 and ***p < 0.001

Feeding HFD reduced testicular SOD activity by 21% when compared to C group (*p* = 0.003; Fig. 4A). Supplemented HFD-fed rats had 30% increase in testicular SOD activity as compared to H group (*p* = 0.001; Fig. 4A). Feeding supplemented HFD did not significantly affect testicular catalase activity as shown in Fig. 4B. A significant interaction effect of HFD and supplement (*p* < 0.001) was observed on testicular GPx activity suggesting that HFD-fed rats had 44% reduced GPx activity compared to CD-fed rats (*p* < 0.001; Fig. 4C) as analysed by simple main effect. This effect was normalized by supplementation in HS rats (HS > H, ~ 70%, *p* < 0.001; Fig. 4C). Supplementation in CD did not impact testicular GPx activity (Fig. 4C). Testicular glutathione content was not significantly affected by HFD intake (Fig. 4D). An overall significant effect of supplement (*p* = 0.013) on testicular GSH content indicating that supplementation in HFD appeared to increase testicular glutathione level by 19% when compared to HFD-fed rats (*p* = 0.015, Fig. 4D). Supplementation of control diet did not significantly affect the testicular glutathione level (CS vs C *p* = 0.6, Fig. 4D).

### Effect of HFD and supplementation on testicular susceptibility index of pro-oxidative damage

**Fig. 5:**
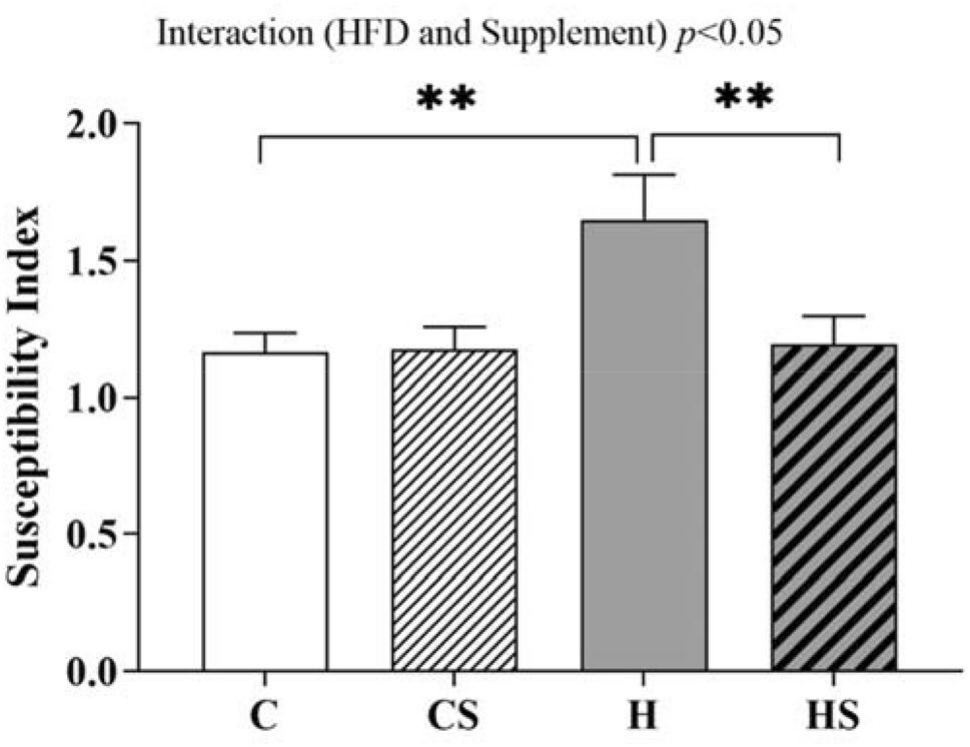
Testicular susceptibility index of pro-oxidative damage. Data are shown as mean ± SEM (*n* = 11–12 per group). Effects of HFD and supplement were assessed by simple main effect analysis, and the resulting post hoc was considered. HFD and supplement interaction effect *p* < 0.05. *C* control diet, *CS* control diet with supplement, *H* HFD, *HS* HFD with supplement. **p < 0.01

The testicular susceptibility index of pro-oxidative damage was calculated from the enzymatic activity of SOD, catalase and GPx as desribed in the method section. A significant interaction effect of HFD and supplement was observed on testicular susceptibility index of pro-oxidative damage indicating that HFD-fed rats had 42% increase in susceptibility index of pro-oxidative index compared to CD-fed rats (*p* = 0.003; Fig. 5). Supplemented HFD-fed rats had 28% reduced susceptibility index when compared to HFD-fed rats (*p* = 0.006; Fig. 5). No difference in the susceptibility index was observed in CS rats (CS v C; *p* > 0.05) (Fig. 5).

### Correlation of testicular susceptibility index with sperm and testicular oxidative stress parameters

To further confirm how the testicular susceptibility index reflects other oxidative stress markers of sperm and testis, we correlated the index with sperm and testicular oxidative stress parameters. Correlational analyses (Table 3) indicated that the testicular susceptibility index correlated significantly with the measures of sperm and testicular oxidative damage. Specifically, testicular susceptibility index positively correlated with sperm oxidative DNA damage (*r* = 0.55, *p* < 0.0001, Table 3), testicular oxidative DNA damage (*r* = 0.31, *p* = 0.03, Table 3) and oxidative lipid damage (*r* = 0.31, *p* = 0.03, Table 3). A weak negative correlation was also observed between susceptibility index and GSH content in testis (*r* = −0.2, *p* = 0.07).

**Table 3:** Correlations between testicular susceptibility index (SI) and oxidative stress parameters in sperm and testis

	Testicular SI	
	*r*	*p*
Sperm 8-OHdG content	0.55	< 0.0001
Testis 8-OHdG content	0.31	0.03
Testis MDA content	0.31	0.03

Data are presented as scatterplots of individual values and analysed by Pearson correlations, *n* = 47

### Correlation of folate content with GSH content in testis

To investigate how micronutrient supplement impacts testicular GSH content, we correlated the testicular folate content (an important component of micronutrient supplement) with testicular GSH content. Correlational analysis indicated that the testicular folate content positively correlated with testicular GSH content (*r* = 0.42, *p* < 0.01, Supp Fig. 2).

### Effect of HFD and supplementation on testicular gene expression involved in oxidative stress

Feeding HFD did not significantly alter mRNA expression of Nrf2, NFκB-p65, IL-6, IL-10 and TNF**-**α in testis (Table 4). No significant effect of supplementation was observed on mRNA expression of NFκB-p65, IL-6, IL-10 and TNF**-**α. Interestingly, an overall significant effect of supplementation to increase expression of Nrf2 gene was found, however, no between group differences were found to be significant in the post hoc analysis (Table 4).

**Table 4:** Effect of HFD and supplement on testicular mRNA expression of genes associated with oxidative stress in rats

Relative expression	C	CS	H	HS	HFD effect	Supplement effect	Interaction effect
Nrf2	1.0 ± 0.04	1.10 ± 0.05	0.94 ± 0.05	1.06 ± 0.04	0.6	**0.018**	0.556
NFκB-p65	1.0 ± 0.04	0.96 ± 0.04	1.02 ± 0.05	1.0 ± 0.05	0.448	0.471	0.864
IL-6	1.0 ± 0.11	0.96 ± 0.04	1.07 ± 0.11	0.99 ± 0.07	0.678	0.608	0.725
IL-10	1.0 ± 0.14	1.07 ± 0.13	0.92 ± 0.14	1.04 ± 0.09	0.691	0.468	0.849
TNF-α	1.0 ± 0.11	1.0 ± 0.09	1.17 ± 0.07	1.02 ± 0.06	0.280	0.389	0.362

Data are shown as mean ± SEM (n = 11–12 per group). Relative gene expression was calculated using the 2^−ΔΔ*C*T^ method, normalised against two reference genes (Gapdh and TBP) and an external calibrator using pooled sample. Effects of HFD and supplement were assessed by two-way ANOVA. Differences were considered significant at *p* < 0.05 (Bolded)

*C* control diet, *CS* control diet with supplement, *H* HFD, *HS* HFD with supplement, *Nrf2* nuclear factor erythroid 2-related factor 2, *NFκB-p65* nuclear factor kappa-light-chain-enhancer of activated B cells, subunit p65, *IL-6* interleukin-6, *IL-10* interleukin-10, *TNF-α* tumor necrosis factor alpha, *Gapdh* glyceraldehyde 3-phosphate dehydrogenase, *TBP* TATA-box-binding protein

## Discussion

In this study, rats fed HFD for 27.5 weeks had 21% increase in total body weight (96% increase in retroperitoneal fat) and 17% decrease in relative testicular weight compared to control fed rats. The HFD-fed rats had a significant increase of 24% and 40% 8-OHdG content in their sperm and testis, respectively. This oxidative DNA damage was also associated with 21% decrease in SOD activity and 44% decrease in GPx activity in HFD-fed rat testis. Furthermore, when we calculated the susceptibility index of pro-oxidative damage as described by Peltola et al. [36], HFD-consuming rats appeared to be more vulnerable to oxidative damage. It is likely that the unaltered sperm count observed in our HFD-fed rats did not exclude further possible detrimental impact of energy dense food on sperm and testis in agreement with a recent rodent study, where mice consuming HFD for 200 days developed irreversible deteriorated sperm motility, viability, morphology and testicular metabolites related to antioxidant activity without affecting sperm count [41]. Strikingly, our micronutrient supplement containing folic acid, zinc, vitamin B_6_, choline and betaine, when combined with obesogenic diet, was able to normalise body weight (and adiposity), testis weight, sperm and testicular oxidative DNA damage, testicular susceptibility index in association with SOD and GPx activity. In addition, micronutrient supplementation had an impact to increase testicular glutathione and folate content, Nrf2 gene expression and reduce lipid peroxidation. To our knowledge, this is the first intervention study where antioxidant-based micronutrient supplementation in rodents was able to ameliorate HFD-induced oxidative damage in both sperm and testis.

Several rodent studies reported similar sperm and/or testicular oxidative damage as well as testicular weight upon feeding HFD [3, 5, 6, 42–46] as we have demonstrated here. However, limited work has examined the effects of supplementation with the micronutrients used in this study (single or combined) on HFD-induced oxidative damage in both sperm and testis. In a recent rodent study by McPherson et al., mice were fed HFD for 10 weeks followed by 12 days micronutrient intervention containing folic acid 1.5 mg/kg, zinc 61 mg/kg, vitamin C 700 mg/kg, vitamin E 78 mg/kg, lycopene 0.3 mg/kg, selenium 0.44 mg/kg and green tea extract 0.95 mg/kg. The supplemented HFD-fed mice had reduced ROS and 8-OHdG concentration in sperm suggesting that micronutrient supplementation only for 12 days was able to reduce oxidative damage in the sperm of obese mice. However, they did not investigate the potential effects of their supplement on oxidative damage in mouse testis [5]. The 12-day intervention designed by McPherson et al., was intended to target sperm oxidative damage during epididymal transit [5], however, this is not adequate exposure to cover a full cycle of spermatogenesis (35 days) in mice [47]. In males, one might expect intervening before conception over at least a full cycle of spermatogenesis would be required to reduce sperm oxidative damage or to facilitate repair from previous damage. On the other hand, in our study, rats were supplemented from weaning throughout their life span in line with the availability of high caloric and nutrient poor food as a key contributor of global obesity [48] and the resulting prevalence of childhood obesity [49]. Therefore, increasing the duration of an intervention could be an ideal approach to target obesity-induced oxidative damage throughout the spermatogenesis process. Considering the full cycle of spermatogenesis (54 days) in rats, an intervention study was conducted using a different micronutrient supplement from our formulation. In that study (48), micronutrient supplementation (vitamin E 2 g/kg, vitamin C 2 g/kg and astaxanthin 0.6 g/kg) for 12 weeks in HFD-fed Wistar rats did not appear to improve histological changes in testis. However, the potential effects of this supplementation on sperm and testicular oxidative DNA damage were not investigated [50].

Testicular cells and sperm are vulnerable to ROS attack, lipid peroxidation and oxidative DNA damage due to high content of polyunsaturated fatty acid in plasma membrane of testicular cells and sperm [51], a limited amount of cytoplasm (the reservoir of antioxidant) in sperm [52] and the presence of ROS producing enzymes in both sperm and testis [53–55]. To combat this oxidative attack, the testicular cells possesses a wide range of enzymatic and nonenzymatic antioxidants including SOD, catalase, GPx, and GSH [56]. SOD, catalase and GPx are considered first-line enzymatic antioxidants as they are the first to neutralize superoxide radicals [57]. Superoxide radical is the most readily formed free radical due to high consumption of mitochondrial oxygen during spermatogenesis in testis [51] or phagocytosis of germ cell debris in testis by testicular somatic cells, producing superoxide radical by-products [58]. The superoxide radical is rapidly converted to H_2_O_2_ in the presence of SOD. However, the accumulated H_2_O_2_ can induce oxidative damage to lipids, proteins and DNA [37]. In addition, this accumulated H_2_O_2_ in the presence of Fe^2+^ can be converted to another toxic free radical, hydroxyl radical (*OH) through Fenton reaction [57]. Therefore, to protect biological systems from H_2_O_2_-induced oxidative damage, H_2_O_2_ needs to be eliminated by either catalase or predominantly by glutathione-dependent enzyme, GPx in testis [36]. In a rat study, it has been shown that epididymal sperm and the developing sperm in testis has high SOD activity associated with low glutathione and glutathione-dependent enzymatic antioxidant activity particularly GPx [56]. This difference between SOD and GPx activity may lead to saturation of the protective systems against H_2_O_2_ which may further induce oxidative damage as described using susceptibility index of pro-oxidative damage. Moreover, the positive correlation of testicular susceptibility index with sperm 8-OHdG content, testicular 8-OHdG and MDA content observed in our study suggest that impaired first-line enzymatic antioxidant activity in testis could reflect oxidative DNA damage in both sperm and testis. Therefore, it is critical to improve testicular first-line antioxidant defence systems to target obesity-induced oxidative damage in male reproductive system. Our study is the first to investigate the beneficial effects of micronutrient supplement to mitigate HFD-induced impaired testicular first-line antioxidant activity.

Testicular MDA, and glutathione content, catalase activity and expression of genes involved in oxidative stress were not significantly affected by the HFD feeding protocol in the current study. This is in contrast to previous studies reporting increased MDA content, expression of NFκB-p65, IL6 and TNF-α gene, decreased glutathione content, catalase activity and expression of Nrf2 and IL10 gene in testis of HFD-fed rodents [3, 4, 42–44]. However, the investigation of mRNA and protein expression of Nrf2, NFκB-p65, IL6, IL10 and TNF-α in seminal plasma would be of future interest given the altered cytokine levels in seminal plasma induced by HFD in a recent study [59]. Nrf2 and NFκB-p65 are the two key transcription factors regulating cellular responses to oxidative stress. Under normal physiological conditions, both transcription factors remain in harmony [60]. Nrf2 binds to antioxidant responsive elements (ARE) in the promoters of its target genes and upregulates the gene expression of antioxidants including SOD, catalase, GPx, GSH which effectively neutralizes free radicals and inactivates NF-κB-p65 [60, 61]. However, in the event of excess ROS production leading to oxidative stress, NF-κB-p65 activation further increases the production of ROS and inflammatory cytokines including IL-1b, IL-6, TNF-α and downregulates Nrf2 [60, 61]. It is well established that increased body weight is associated with impaired sperm quality and increased oxidative damage in reproductive system [62]. Therefore, the lack of difference observed in our study may be due to a modest 21% body weight difference between obese and lean animals whereas other studies reported 30–50% body weight difference was associated with increased oxidative stress and decreased antioxidant capacity in male reproductive system [4, 42].

No significant effect of HFD was also seen in our study on testicular folate content. The effect of HFD-induced obesity on testicular folate content is relatively unknown. No previous study has investigated the effects of HFD on testicular folate content in rodents. On the other hand, in this study, an increased testicular level of folate (one of the key micronutrients in our supplement) was observed in supplemented rats regardless of their diet. It is evident that folate has potential to protect biological systems from oxidative damage [63]. Therefore, high level of testicular folate upon supplementation is likely to improve its antioxidant capacity. Interestingly, our supplement had an impact to increase testicular glutathione level, expression of the Nrf2 gene and decrease MDA content. Being a major cellular antioxidant, glutathione plays a key role in protecting cells against oxidative damage [64]. No study has previously reported the effects of supplementation containing our micronutrients (single or combined) to improve testicular glutathione level in HFD-fed rodents. Moreover, a significant positive association between testicular folate and glutathione level was seen in our study. This suggests that increased testicular folate level resulting from our supplementation has an impact to drive the one-carbon intermediate, homocysteine towards trans-sulphuration pathway leading to glutathione formation and thus promotes associated antioxidant activity.

Interestingly, our micronutrient supplement did not appear to reduce sperm oxidative DNA damage in rats consuming control diet. Similarly, the study by McPherson et al., where CD-fed mice were given micronutrient containing folic acid, zinc, vit C, vit E, lycopene, selenium and green tea extract for 12 days did not show any change in sperm oxidative DNA damage [5]. It could be conceivable that micronutrient supplement may be of most benefit to individuals who are micronutrient deficient and thus counteract the complications associated with micronutrient deficiencies. However, in the context of testicular oxidative damage and folate content, we observed that supplemented control fed rats had reduced testicular 8-OHdG and increased folate content suggesting some beneficial effects of our supplementation in lean animals that are not challenged by HFD. Overall, given the increased prevalence of male obesity and potential micronutrient deficiency, this study strongly supports the beneficial effect of our micronutrient supplement in interrupting obesity-induced sperm and testicular oxidative damage.

## Conclusion

Our study is the first to demonstrate that HFD-induced impairment of testis first line of antioxidant defence, particularly SOD and GPx activity, had a significant impact to increase sperm and testicular oxidative DNA damage in rodents. Our antioxidant property-based micronutrient supplement has potential to interrupt HFD-induced oxidative DNA damage in both sperm and testis by improving testicular expression of Nrf2 gene, glutathione content and associated antioxidant activity. This is the first work assessing the impact of a unique micronutrient supplement combining folate, vitamin B_6_, choline, betaine, and zinc on testicular and sperm oxidative stress in rats. Such findings could provide an opportunity to utilize micronutrient-based supplement for ameliorating obesity-induced paternal programming of offspring health. Further study is warranted to decipher the mechanism(s) underpinning the beneficial effects of our micronutrient supplement.

## Supplementary Information

Below is the link to the electronic supplementary material.

Supplementary file1 (DOCX 170 KB)

Supplementary file2 (DOCX 76 KB)

Supplementary file3 (DOCX 15 KB)

## Abbreviations

ARE: Antioxidant responsive elements
BWW: Biggers, Whitter and Whittingham
CD: Control diet
CS: Control diet containing micronutrient supplement
CVs: Coefficient of variations
GPx: Glutathione-peroxidase
GSH: Glutathione
HFD: High-fat-diet
H_2_O_2_: Hydrogen peroxide
HS: HFD-containing micronutrient supplement
IL-6: Interleukin-6
IL-10: Interleukin-10
MDA: Malondialdehyde
Nrf2: Nuclear factor erythroid 2-related factor 2
NFκB-p65: Nuclear factor kappa-light-chain-enhancer of activated B cells, subunit p65
SI: Susceptibility index
SOD: Superoxide-dismutase
TNF-α: Tumor necrosis factor alpha
ROS: Reactive oxygen species
8-OHdG: 8-Hydroxy-2-deoxyguanosine
