# Supplementary material for: Ameliorating high-fat diet-induced sperm and testicular oxidative damage by micronutrient-based antioxidant intervention in rats
Source: Eur J Nutr. 2022 Jun 16;61(7):3741–53. doi: 10.1007/s00394-022-02917-9 (PMC9464124; doi:10.1007/s00394-022-02917-9)
Supplement: Supplementary file 1 — Supplementary file1 (DOCX 170 KB) [file 394_2022_2917_MOESM1_ESM.docx]

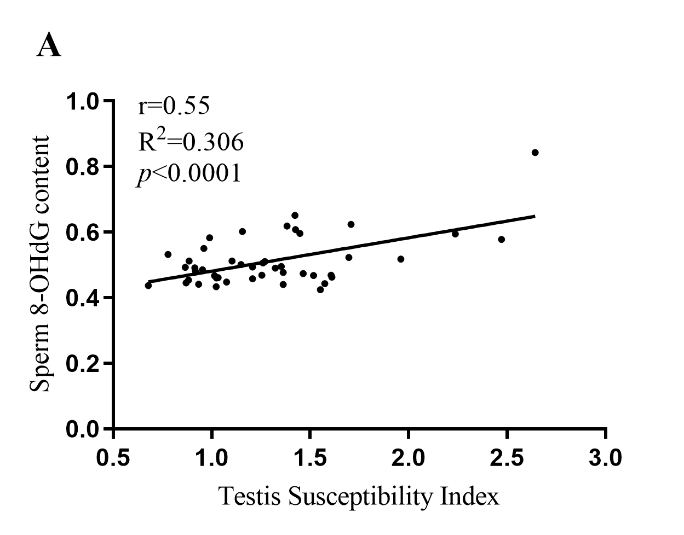

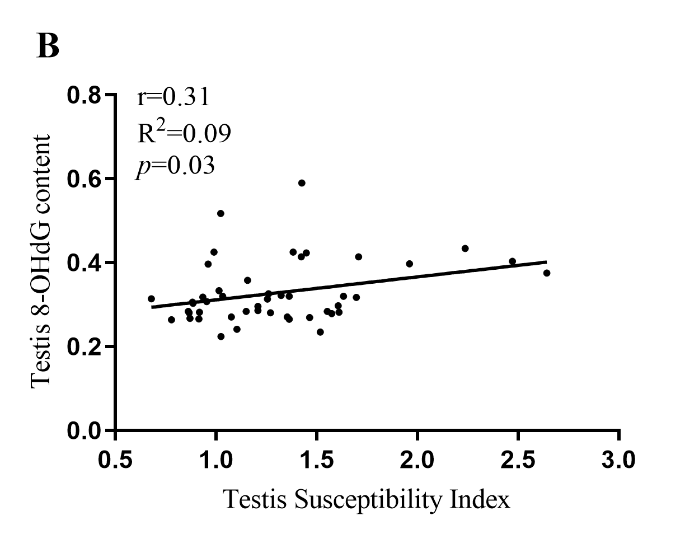

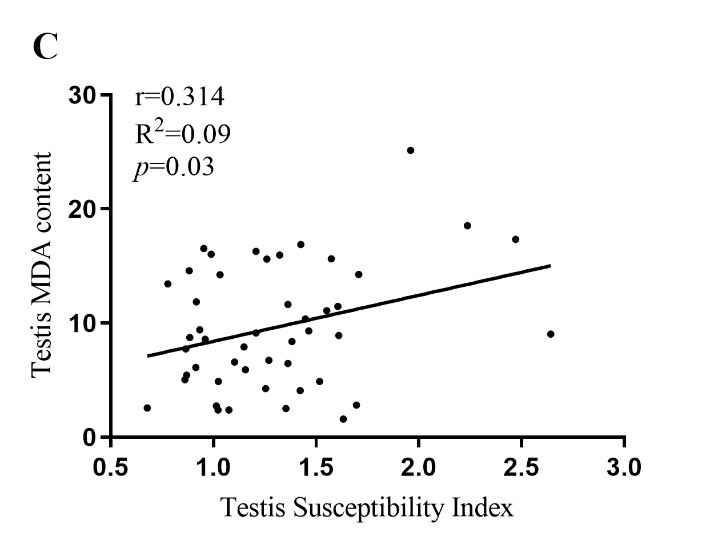


**Supplementary Fig. 1** **Correlations between testicular susceptibility index and oxidative stress parameters in sperm and testis.** (**A**) Correlation between testicular susceptibility index and sperm 8-OHdG content (**B**) correlation between susceptibility index and 8-OHdG content in testis and (**C**) correlation between susceptibility index and MDA content in testis. Data are presented as scatterplots of individual values and analysed by Pearson correlations, n = 47.
