# Supplementary material for: Ameliorating high-fat diet-induced sperm and testicular oxidative damage by micronutrient-based antioxidant intervention in rats
Source: Eur J Nutr. 2022 Jun 16;61(7):3741–53. doi: 10.1007/s00394-022-02917-9 (PMC9464124; doi:10.1007/s00394-022-02917-9)
Supplement: Supplementary file 2 — Supplementary file2 (DOCX 76 KB) [file 394_2022_2917_MOESM2_ESM.docx]

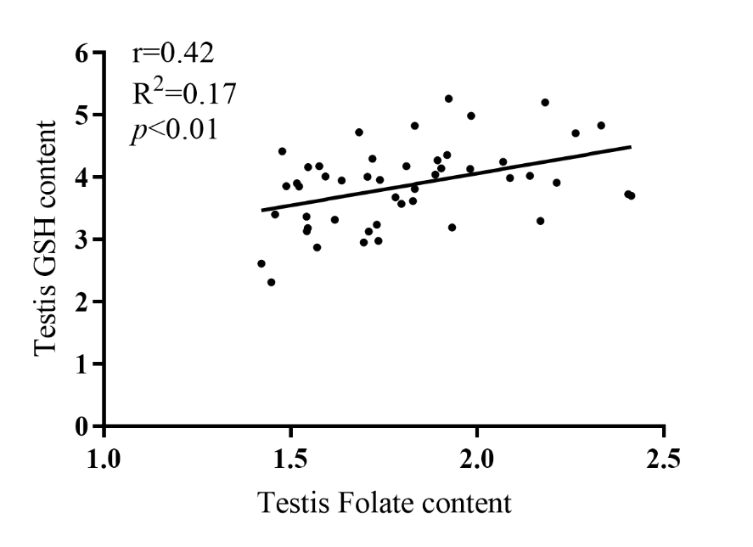


**Supplementary Fig. 2** **Correlation between folate and GSH content in testis**

Data are presented as scatterplots of individual values and analysed by Pearson correlations, n = 47.
