# Supplementary material for: Ameliorating high-fat diet-induced sperm and testicular oxidative damage by micronutrient-based antioxidant intervention in rats
Source: Eur J Nutr. 2022 Jun 16;61(7):3741–53. doi: 10.1007/s00394-022-02917-9 (PMC9464124; doi:10.1007/s00394-022-02917-9)
Supplement: Supplementary file 3 — Supplementary file3 (DOCX 15 KB) [file 394_2022_2917_MOESM3_ESM.docx]

**Supplementary Table 1 Genes selected for RT-qPCR in the testis.**

|  | Gene name | Applied Biosystems assay ID |
| --- | --- | --- |
| Reference gene | B2M: Beta-2-microglobulin | Rn00560865_m1 |
|  | B-actin: Beta actin | Rn00667869_m1 |
|  | HPRT-1: Hypoxanthine phosphoribosyl-transferase 1 | Rn01527840_m1 |
|  | Gapdh: Glyceraldehyde 3-phosphate dehydrogenase | Rn01749022_g1 |
|  | TBP: TATA-box-binding protein | Rn01455646_m1 |
| Gene of interest | Nrf2: Nuclear factor erythroid 2-related factor 2 | Rn00477784_m1 |
|  | NFκB-p65: Nuclear factor kappa-light-chain-enhancer of activated B cells, subunit p65 | Rn01502266_m1 |
|  | IL-6: Interleukin-6 | Rn01410330_m1 |
|  | IL-10: Interleukin-10 | Rn00563409_m1 |
|  | TNF-α: Tumor necrosis factor alpha | Rn99999017_m1 |
